# β‐Cyclodextrin/eucalyptol inclusion complex for improved postharvest date moth control

**DOI:** 10.1002/ps.8977

**Published:** 2025-06-13

**Authors:** Hadhami Chargui, Miriana Kfoury, Abir Soltany, Tasnim Djebbi, Soumaya Haoual Hamdi, Sophie Fourmentin, Jouda Mediouni Ben Jemâa

**Affiliations:** ^1^ Faculty of Sciences of Bizerte University of Carthage Tunis Tunisia; ^2^ Laboratory of Biotechnology Applied to Agriculture, National Agricultural Research Institute of Tunisia (INRAT) University of Carthage Tunis Tunisia; ^3^ Université Littoral Côte d'Opale, UR 4492, UCEIV, Unité de Chimie Environnementale et Interactions sur le Vivant Dunkerque France

**Keywords:** biopesticide, cyclodextrin, natural product, *Ectomyelois ceratoniae*, fumigation, release

## Abstract

**BACKGROUND:**

The threats posed by pesticides to the environment and human health are well recognized by the scientific community. Thus, there is a need to go beyond their use and explore new alternatives. This study aims to evaluate the potential of eucalyptol, an organic natural compound found in certain plants essential oils, to control the date moth, *Ectomyelois ceratoniae.* However, eucalyptol is volatile and can be lost during application, so it has been encapsulated in cyclodextrins (CDs) to overcome this drawback.

**RESULTS:**

Retention and formation constants of CD/eucalyptol inclusion complexes were determined. Solid inclusion complexes were then prepared in the form of powder or pellets using different (β‐CD/eucalyptol) molar ratios. Release studies were performed for powder and pellets of inclusion complexes either placed in a humidity‐controlled environment or immersed in water. The fumigant insecticidal activity of free and encapsulated eucalyptol was evaluated against the fifth instar larvae of *Ectomyelois ceratoniae*. Results showed that CD efficiently encapsulate eucalyptol in solution and in solid state with the 1:1 molar ratio showing the best encapsulation efficiency (79%) and loading capacity (135 mg/g). Under all conditions, eucalyptol was released more slowly from pellets. Higher fumigant activity, adult emergence inhibition and persistence were achieved by immersing solid powder and pellets of inclusion complex in water.

**CONCLUSION:**

The findings showed that CD provided a controlled release of eucalyptol with improved fumigation efficacy against date moth. Encapsulation of CD therefore appears to be a valuable strategy for developing biopesticides with reduced loss through regulated release and prolonged activity. © 2025 The Author(s). *Pest Management Science* published by John Wiley & Sons Ltd on behalf of Society of Chemical Industry.

## Graphical abstract

Powder or pellets of CD/eucalyptol solid inclusion complex immersed in water showed promising efficacy in controlling fifth instar larvae of *Ectomyelois ceratoniae*. Therefore, this strategy should be considered for the development of non‐toxic natural pesticides.

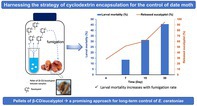

## 1 INTRODUCTION

*Ectomyelois ceratoniae* Zeller 1881 (Lepidoptera: Pyralidae) is a highly destructive polyphagous pest and a major phytosanitary problem for the date palm industry.^1^ *Ectomyelois ceratoniae* often infests dates in the field during the ripening stage and remains in infested crops during postharvest processing, becoming a pest of the stored produces. Pyralidae infestation reduces the quality and commercial value of dates, resulting in significant economic losses to producers. Among the various pest control methods (i.e., physical control, mass trapping, biological control, reproductive control and so on), the primary technique for controlling moths remains the chemical control using synthetic pesticides. Commodity fumigation is the most efficient and scalable method of controlling insects in stored products using volatile chemicals. However, the global commitment to phase out chemical substances with adverse effects on health, the environment and the ozone layer has led to the search of safer and more environmentally friendly product alternatives such as natural plant metabolites.^2^ Essential oils represent a promising source of biopesticides.^3^

**Figure 1:**
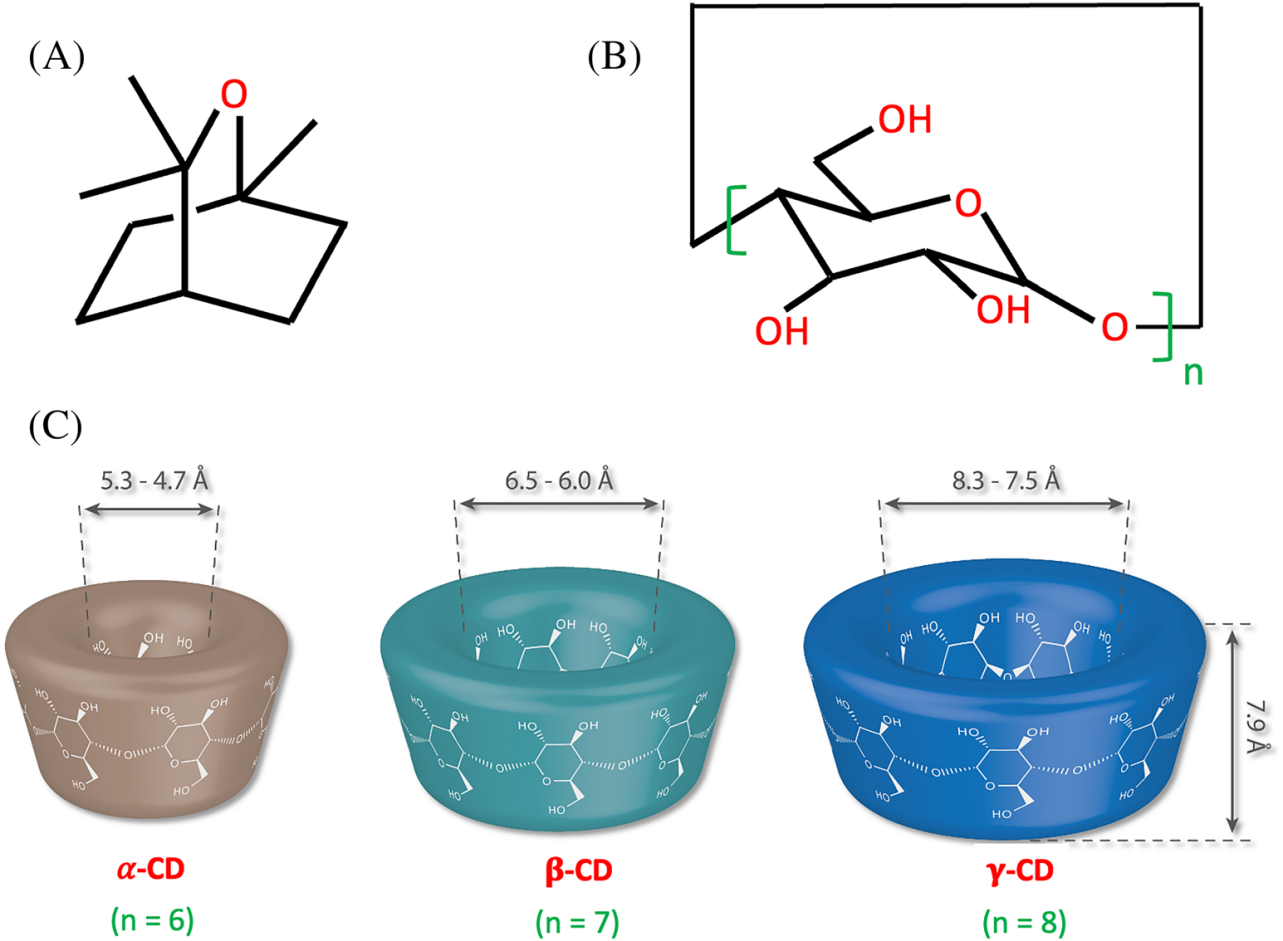
(A) Chemical structure of eucalyptol and (B) native cyclodextrins (CDs). (C) Representation of the geometry and dimensions of α‐CD, β‐CD and γ‐CD.

Eucalyptol (1,8‐cineole) (Fig. 1) is an aromatic compound found in the essential oils of various plants such as *Eucalyptus globulus*, *Rosmarinus officinalis* and *Laurus nobilis*.^4^, ^5^ It has been shown to exhibit different biological activities, including antimicrobial and antifungal effects,^6^ as well as anticancer, anti‐inflammatory and antioxidant activities.^7^, ^8^ In addition, eucalyptol has the ability to act as an insect repellent, providing protection against parasites such as mosquitoes, scabies mites and other organisms.^9^ As a result, the use of this natural bioactive compound as an environmentally friendly alternative to synthetic pesticides has attracted considerable interest.^10^, ^11^, ^12^ Researchers have investigated the ability of eucalyptol to control harmful insects. It has been shown to be effective against *Sitophilus oryzae* and *Tribolium castaneum* ^13^ as well as *Plutella xylostella*.^14^ The toxic effect of this natural terpene against the date moth *Ectomyelois ceratoniae* was also demonstrated.^15^ However, the interesting properties of eucalyptol cannot hide the drawback of high volatility associated with its use. Due to its volatility, eucalyptol may have limited persistence under field conditions or when used as postharvest treatment of crops.^16^, ^17^ Thus, encapsulation might be a promising strategy to address this challenge.^18^, ^19^

Molecular encapsulation in cyclodextrins (CDs) is one of the most efficient technologies for the retention of bioactives.^20^, ^21^ CDs are cyclic oligosaccharides composed of α‐1,4‐linked glucose units. Native CDs forming six, seven, and eight glucose residues are called α‐CD, β‐CD and γ‐CD, respectively^22^ (Fig. 1). CDs are industrially produced from renewable sources, such as corn or potato starch, making them readily available, sustainable and environmentally friendly. Moreover, native CDs are GRAS (generally recognized as safe) and have been included in the European list of food additives as E‐457 (α‐CD), E‐458 (γ‐CD) and E‐459 (β‐CD).^22^ CDs possess a unique structure with a hydrophilic surface and a hydrophobic cavity. The latter provides the ability to accommodate hydrophobic compounds to form inclusion complexes. In particular, CDs can encapsulate guests for application in both liquid and solid formulations. Encapsulation in CDs generally improves the properties of guests, namely their apparent solubility and stability. In addition, encapsulation in solid CD complexes allows the development of formulations that are easy to store, handle and dose, and ensure, under controlled conditions, the sustained release of volatile guests essential for efficient biological activity.^23^, ^24^ Encapsulation of eucalyptol would therefore achieve a stronger and more persistent insecticidal effect. It could also provide a more acceptable dosage form for the end users. To validate this hypothesis, the efficacy of β‐CD and γ‐CD to encapsulate eucalyptol was first evaluated. Then, solid inclusion complexes were prepared using different β‐CD/eucalyptol molar ratios via the co‐precipitation coupled to the lyophilization method. In order to determine the optimal application conditions, different solid dosage forms (i.e., powder or pellet) as well as testing parameters (i.e., immersion in water, temperature and humidity conditions) were evaluated for their ability to ensure sustained release of eucalyptol as well as larvicidal fumigant toxicity and adult emergence inhibition against the fifth instar larvae of *Ectomyelois ceratoniae*. The long‐term persistence of these treatments was also evaluated. The findings of these investigations will help to open up a promising route for developing natural pesticides treatments based on CD/natural volatiles inclusion complexes and to standardize the handling and application of these natural active materials.

## 2 MATERIALS AND METHODS

### 2.1 Chemical materials

Eucalyptol was purchased from Sigma Aldrich (Taufkirchen, Germany). CDs (β‐CD and γ‐CD) were provided by Wacker‐Chemie (Lyon, France). Potassium sulfate (K_2_SO_4_) was purchased from Acros Organics (Geel, Belgium). Distilled water was used throughout this work.

### 2.2 Characterization of CD/eucalyptol inclusion complexes in solution

#### 2.2.1 Static headspace coupled to gas chromatography

Briefly, 10 mL of aqueous CD solutions of various concentrations (0.5, 1, 2 and 4 mm) were placed in 22 mL glass vials. An appropriate amount of eucalyptol (10 ppm) was then added. Vials were sealed and allowed to equilibrate at 30 °C before analysis. Measurements were performed using a Thermo Scientific TriPlus™ 500 Headspace autosampler coupled to a TRACE™ 1300 series gas chromatograph equipped with a flame ionization detector and a DB624 column using nitrogen as the carrier gas (Thermo Fisher Scientific, Waltham, MA, USA). Gas chromatography (GC) settings were set as follows: column temperature 120 °C and GC cycle of 8 min.

##### 2.2.1.1 Retention studies

The retention capacity of the studied CDs (β‐CD and γ‐CD) towards eucalyptol was expressed using the following equation:

(1) $$\text{Retention}\;\left(\%\right)=\left(1-\frac{A_{\mathrm{CD}}\;}{A_{0}}\right)\times \;100$$

where *A*_0_ and *A*_CD_ stand for the peak areas of eucalyptol in the absence and the presence of CD, respectively. All experiments were done in triplicate.

##### 2.2.1.2 Determination of formation constant (*K*_f_)

The formation constant (*K*_f_) of inclusion complexes of eucalyptol with β‐CD and γ‐CD were determined at 30 °C using the static headspace gas chromatography (SH‐GC) titration method.^25^ Vials were prepared and treated as indicated in Section 2.2.1 An algorithmic treatment was used to calculate *K*_f_ values.

### 2.3 Preparation of β‐CD/eucalyptol solid inclusion complexes (powder and pellet)

The β‐CD/eucalyptol solid inclusion complexes were prepared at three molar ratios (1:2, 1:1 and 2:1) using the co‐precipitation coupled to freeze‐drying method. Appropriate amounts of eucalyptol were added dropwise to an aqueous solution of β‐CD (10^−2^ m) previously heated at 40 °C. Heating was then stopped and the mixture was stirred at 150 rpm for 5 h at room temperature and then kept at 4 °C for 48 h. The precipitates were then recovered by vacuum filtration, frozen and lyophilized (−85 °C, 0 Pa) using a Christ Alpha 2‐4 LD Freeze‐dryer (Martin Christ Gefriertrocknungsanlagen GmbH, Germany) until water was completely removed. The recovered material was considered as β‐CD/eucalyptol solid inclusion complex in powder form. A fraction of this powder was compressed using a powder compressing device (Powder Press; Specac, USA) with a die and two flat face punches to obtain β‐CD/eucalyptol solid inclusion complex in pellet form.

### 2.4 Determination of loading capacity and encapsulation efficiency

The amount of eucalyptol in the solid inclusion complexes obtained using different β‐CD/eucalyptol molar ratios (1:2, 1:1 and 2:1) was quantified by using multiple headspace extraction (MHE) coupled to GC using the external standard method.^26^ Briefly, 5 mg of solid inclusion complex or a known amount of eucalyptol (0.1 mg) were dissolved in 10 mL of water and placed in 22 mL sealed vials. All vials were equilibrated at 60 °C then submitted to ten successive extractions and analysis of their headspace at 1‐h time interval. GC settings were set as described in Section 2.2.1.

The loading capacity (LC) and encapsulation efficiency (EE) were expressed as follows:

(2) $$\mathrm{LC}=\frac{{\text{Eucalyptol}}_{\exp}\;\left(\mathrm{mg}\right)}{\text{Inclusion complex}\;\left(g\right)}$$

(3) $$\mathrm{EE}\;\left(\%\right)=\frac{{\text{Eucalyptol}}_{\exp}\;\left(\mathrm{mg}\right)}{{\text{Eucalyptol}}_{t}\;\left(\mathrm{mg}\right)}\;\times \;100$$

where Eucalyptol_exp_ and Eucalyptol_t_ stand for the amounts of eucalyptol (in milligrams) determined experimentally and theoretically in the inclusion complex (in grams), respectively. All preparations and measurements were done in triplicate.

### 2.5 Release studies

The release of eucalyptol from inclusion complexes in powder or pellets was evaluated under two conditions: in a controlled humidity environment or immersed in water.

#### 2.5.1 Powder and pellets placed in controlled humidity environment

Briefly, 5 mg of β‐CD/eucalyptol inclusion complex in the form of solid powder or pellets were placed in open vials and incubated in storage jars at 60 °C. The relative humidity (RH) was regulated at 96% using saturated solution of K_2_SO_4_. At different time intervals (7, 15, 30, 60 and 90 days), the remaining quantity of eucalyptol in the powder or pellets was determined by MHE as described in Section 2.4.

#### 2.5.2 Powder and pellets immersed in water

Briefly, 5 mg of β‐CD/eucalyptol inclusion complex in the form of freeze‐dried powder or pellets were immersed in 10 mL of distilled water. The obtained preparations were allowed to volatilize at room temperature (22 °C) for up to 15 days. At different time intervals, the remaining amount of eucalyptol in solution was determined using MHE as described in Section 2.4.

### 2.6 Insecticidal activity

#### 2.6.1 Collection of infested dates

The infested dates were collected in February 2023 from southern Tunisia. A date dissection was performed in search of *Ectomyelois ceratoniae* fifth instar larvae (L_5_). The other larval stages were then placed in transparent plastic boxes in the rearing room under controlled conditions (temperature: 25 ± 1 °C, RH: 70 ± 1% and photoperiod: 12 h:12 h). They were maintained under these conditions until reaching the L_5_ stage for further use.

#### 2.6.2 Determination of lethal concentration of eucalyptol

Fumigation experiments were conducted to determine the median lethal concentration (LC_50_) of eucalyptol against the L_5_. Bioassays were carried out in 1 L sealed glass containers enclosing 20 dates each (each date contained one L_5_). Different concentrations of free eucalyptol namely 25, 50, 75 and 100 μL/L air were deposited on 5 cm Whatman No. 1 filter papers attached to the screw caps of the glass bottles.^27^ Larvae untreated with eucalyptol were considered as the control. All bioassay experiments were performed under controlled rearing conditions (temperature: 25 ± 1 °C, RH: 70 ± 1% and photoperiod: 12 h:12 h). Each condition was replicated three times.

The mortality rates after 7, 15 and 30 days of exposure were calculated using Abbott's formula^28^:

(4) $$\text{Mortality rate}\;\left(\%\right)=\left(1-\frac{\left(N-T\right)}{\left(N-C\right)}\right)\times \;100$$

where *N* is the total number of larvae, *C* stands for the number of dead untreated larvae (control) and *T* is the number of dead treated larvae.

Larval mortality data was then processed using probit analysis in order to determine the value of LC_50_.

The percentage of emerged adults after 30 days was calculated using the following formula^29^:

(5) $$E\;\left(\%\right)=\frac{A}{N}\;\times \;100$$

where *E* is the adult emergence (%), *N* stands for the total number of larvae and *A* is the total number of emerged adults.

#### 2.6.3 Insecticidal efficacy of the inclusion complexes

The fumigation toxicity of both free and encapsulated eucalyptol was determined at the LC_50_ value. These experiments were conducted on the L_5_ stage, as described in Section 2.6.2. Free eucalyptol (concentration equivalent to LC_50_) was deposited on 5 cm Whatman No. 1 filter papers. Solid powder or pellets of inclusion complex (prepared using the 1:1 molar ratio) were placed in open vials either in controlled humidity conditions (96% RH) or immersed in water inside the sealed glass containers. The median lethal time (LT_50_) was determined using the Probit analysis.^30^

#### 2.6.4 Persistence experiments

Persistence experiments were performed by repeating the earlier protocol but maintaining the infested dates for an additional period (up to 60 days). After removing the treated larvae and recording the mortality, new larvae were introduced. The LT_50_ value for each treatment was analyzed using the Probit method.^30^

### 2.7 Statistical analyses

The LC_50_, LT_50_ values and regression parameters were obtained using Probit analysis in SPSS statistical software version 20.0 (IBM Corporation, Armonk, NY, USA). Significant differences between the mean value (*P* ≤ 0.05) were determined using the Tukey's and Duncan's tests. When necessary, data were transformed to the common logarithm to meet assumptions of normality.

## 3 RESULTS AND DISCUSSION

### 3.1 Characterization of CD/eucalyptol inclusion complexes in solution

#### 3.1.1 Retention of eucalyptol

**Figure 2:**
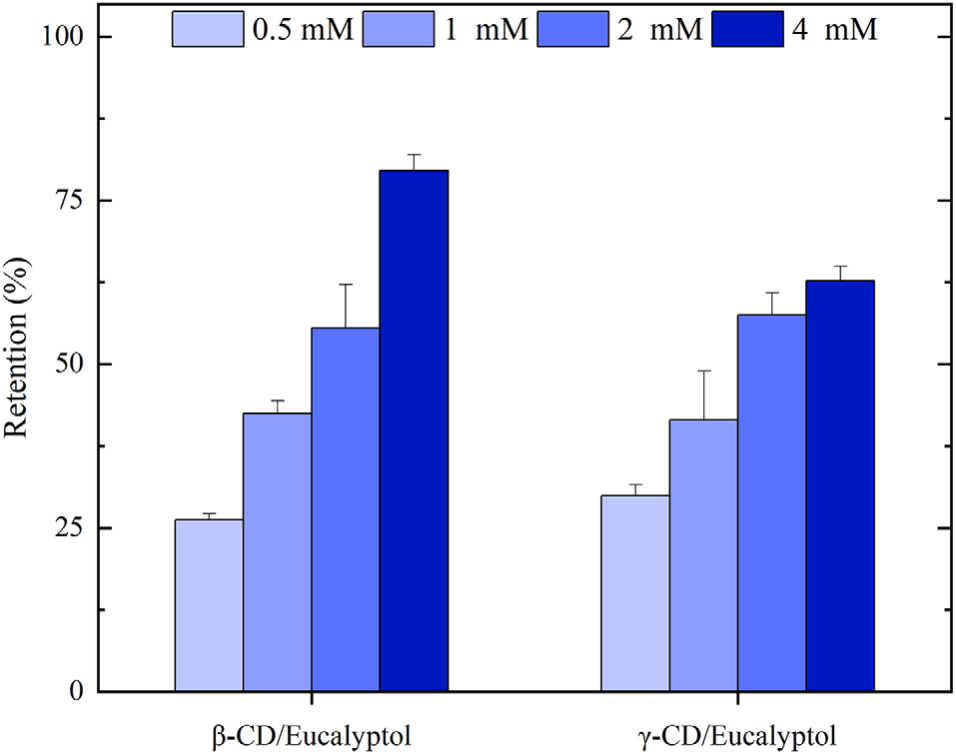
Retention (%) of eucalyptol in β‐CD and γ‐CD at 30 °C.

The ability of native CDs (β‐CD and γ‐CD) to retain eucalyptol was investigated and reported in Fig. 2. Both, β‐CD and γ‐CD efficiently retained eucalyptol. At 0.5 mm CD, 26% and 30% of eucalyptol was retained in solution for β‐CD and γ‐CD, respectively. The retention values increased with increasing concentration of both CDs. This is due to the fact that as more CDs are added, the thermodynamic equilibrium shifts towards the formation of an inclusion complex, resulting in increased eucalyptol retention and thus reduced volatility. The different retention capacities observed for the different CDs suggest differential interactions with eucalyptol due to their different cavity sizes.

#### 3.1.2 Determination of formation constants (*K*_f_)

Formation constant (*K*_f_), also called association constant, is a crucial parameter for measuring the strength of the interaction between a guest and a CD. High *K*_f_ values indicate a strong binding potential of the CD towards the guest molecule and consequently demonstrate significant stability of the resulting inclusion complex. The *K*_f_ values of β‐CD/eucalyptol and γ‐CD/eucalyptol inclusion complexes were determined at 30 °C. They were equal to 625 and 1360 m ^−1^, respectively. These results are consistent with the literature.^31^, ^32^ The lower *K*_f_ value obtained for β‐CD/eucalyptol inclusion complex could be attributed to the bicyclic large structure of eucalyptol (Fig. 1), which increases steric hindrance and reduces its interaction with the smaller cavity of this CD in comparison to the γ‐CD. Thus, the larger cavity of γ‐CD favors the interactions with eucalyptol and showed the best complexation ability towards this guest molecule.

#### 3.1.3 Determination of loading capacity (LC) and encapsulation efficiency (EE)

Solid inclusion complexes were prepared using three β‐CD/eucalyptol molar ratios (1:2, 1:1 and 2:1) via the co‐precipitation coupled to freeze‐drying method.^26^ The amount of eucalyptol in the freeze‐dried complexes was quantified using MHE coupled to GC. Results were expressed as LC and EE (Eqns (1) and (2), respectively) and listed in Table 1.

**Table 1:** Loading capacity (LC) and encapsulation efficiency (EE) values for the solid inclusion complexes prepared at different β‐cyclodextrin (β‐CD)/eucalyptol molar ratios

β‐CD/eucalyptol molar ratio	LC (mg/g)	EE (%)
1:2	133 ± 18	39 ± 05
1:1	135 ± 17	79 ± 10
2:1	115 ± 13	77 ± 08

As can be seen in Table 1, the EE percentage increased as the proportion of β‐CD increased in the preparation medium reaching over 77% and 79% when the inclusion complex was prepared using the 2:1 and 1:1 molar ratios, respectively. These findings are consistent with the literature.^15^, ^33^ The LC was similar for both, 1:2 and 1:1 molar ratios, preparations, while it was lower for the 2:1 molar ratio. A decrease in LC would result in an increase in the formulation bulk to attend the desired concentration of the active compound during application. Thus, a 1:1 molar ratio represents a compromise between the loss of valuable compounds during formulation (EE percentage) and the consideration of bulk volume (LC).

### 3.2 Release studies

Encapsulation in CDs is a reversible process, meaning that the release of the encapsulated guest can be achieved by dissolution of solid complexes or triggered by moisture.^26^ Release studies were performed for solid β‐CD/eucalyptol inclusion complexes, in the form of powder or pellets, either placed at controlled RH or immersed in water.

#### 3.2.1 Powder and pellets placed in controlled humidity environment

**Figure 3:**
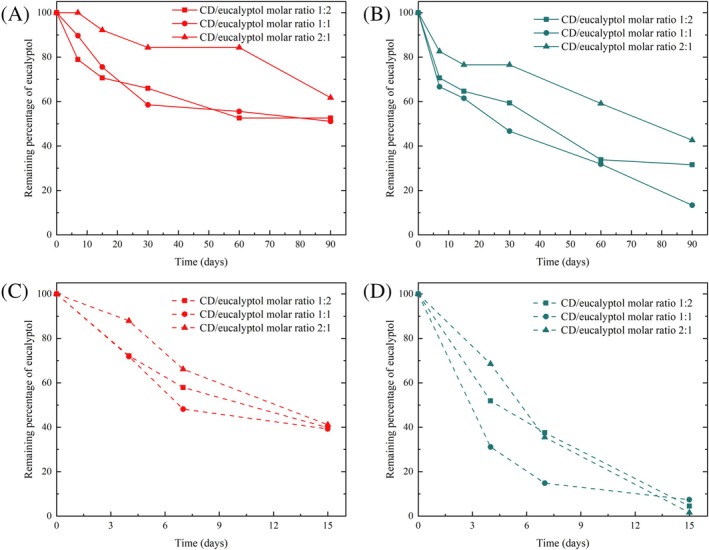
Remaining percentages of eucalyptol for solid inclusion complexes in pellets (A) or powder (B) placed at 60 °C and relative humidity (RH) 96% and for solid inclusion complexes in pellets (C) or powder (D) immersed in water at ambient temperature (22 °C).

Release kinetic experiments were first conducted at 60 °C and 96% RH. At each time interval, up to 90 days, the residual amount of eucalyptol in pellets or powder was determined for all studied molar ratios (1:2, 1:1 and 2:1) and presented in Fig. 3(A),(B).

The results showed that high RH (96%) and elevated temperature (60 °C) can trigger the release of eucalyptol from solid inclusion complex in the powder and pellet form. For all samples, the remaining amount of eucalyptol gradually decreased as the time increased. For all the molar ratios and at all time intervals, the remaining amount of eucalyptol in pellets was higher than in the corresponding powder (for a same molar ratio). This could be due to the fact that powder has a significantly higher surface area than pellets, providing more contact points with moisture, resulting in a more rapid release of eucalyptol than pellets, which require a longer disintegration time.

Regarding the molar ratio, the results showed that the release of eucalyptol from the complex prepared with the 2:1 molar ratio was the slowest in both solid dosage forms. This could be explained by the fact that powder and pellets prepared with this molar ratio contain more CD, resulting in lower release. Powder or pellets prepared using 1:2 and 1:1 molar ratios showed equivalent remaining amounts of eucalyptol at each time interval.

In conclusion, the results showed that elevated temperature (60 °C) and high RH (96%) are able to trigger the release of eucalyptol from solid inclusion complexes. Nevertheless, the release is not complete even after a long period (90 days).

#### 3.2.2 Powder and pellets immersed in water

The β‐CD/eucalyptol solid inclusion complexes in the powder or pellet form were immersed in water, placed in open vials and allowed to volatilize at room temperature (22 °C). At various time intervals, the amount of eucalyptol remaining in the medium was quantified (Fig. 3(C),(D)).

The results showed that when immersed in water, the release of eucalyptol from solid inclusion complexes was faster in powder form than in pellets. After 15 days, the remaining percentage of eucalyptol was around 40% for all pellets. In contrast, there was a complete release of eucalyptol from powder immersed in water for all ratios within the same 15‐day period. This suggests that pellets dissolve in water more slowly than powder. Thus, pelletizing may be an efficient formulation technique to improve powder handling, provide single unit dosage forms and provide sustained release of active volatiles.

### 3.3 Insecticidal activity

#### 3.3.1 Determination of median lethal concentration (LC_50_ ) of eucalyptol

Initially, the fumigant toxicity of free eucalyptol against *Ectomyelois ceratoniae* fifth‐instar larvae (L_5_) was evaluated and the LC_50_ value was determined.

**Figure 4:**
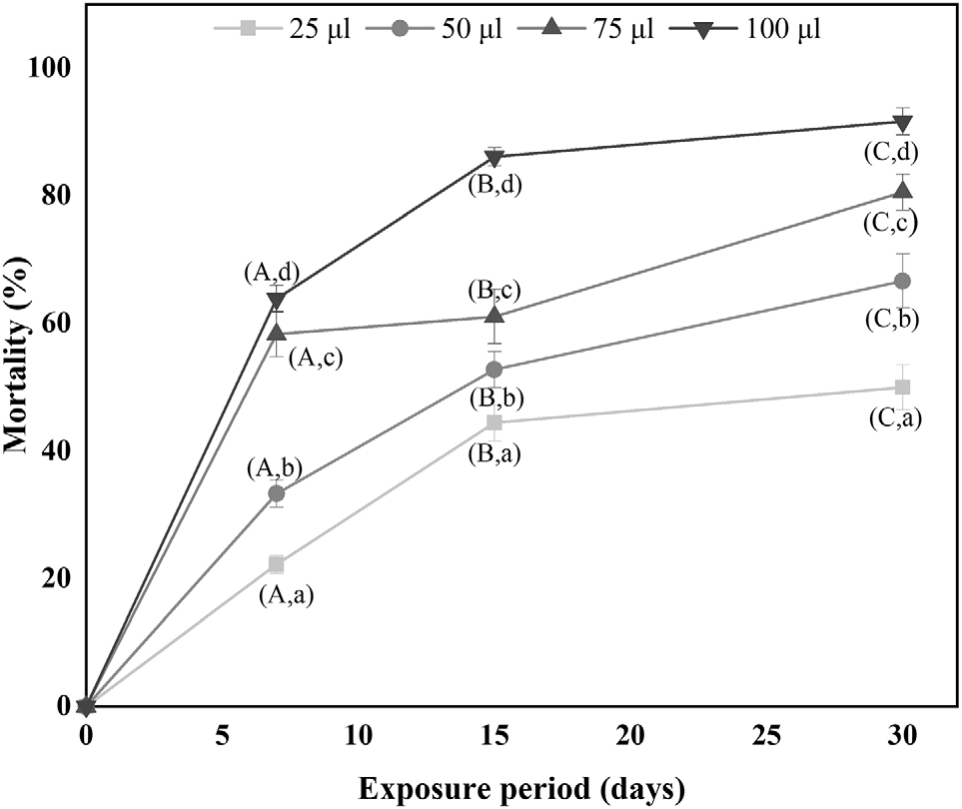
Mortality rates (%) of *Ectomyelois ceratoniae* fifth instar larvae (L_5_) exposed to different concentrations of free eucalyptol (different letters indicate significant difference in percent mortality for each dose (lowercase letters) and exposure time (uppercase letters), Duncan's test at *P* < 0.05).

Figure 4 illustrates the mortality rates after 7, 15 and 30 days of fumigation treatment using different concentrations of free eucalyptol.

The results obviously demonstrated the toxicity of eucalyptol against L_5_ of *Ectomyelois ceratoniae*. The dose‐ and time‐dependent effects were clearly evident. Increasing time and concentration of eucalyptol led to an increase in larval mortality. For example, after 30 days of exposure, mortality increased from 50% to 92% when the concentration of eucalyptol was increased from 25 to 100 μL/L air. The LC_50_ value was determined after 15 days of exposure and was found to be 42 μL/L air.

#### 3.3.2 Evaluation of fumigant activity of free and encapsulated eucalyptol

**Figure 5:**
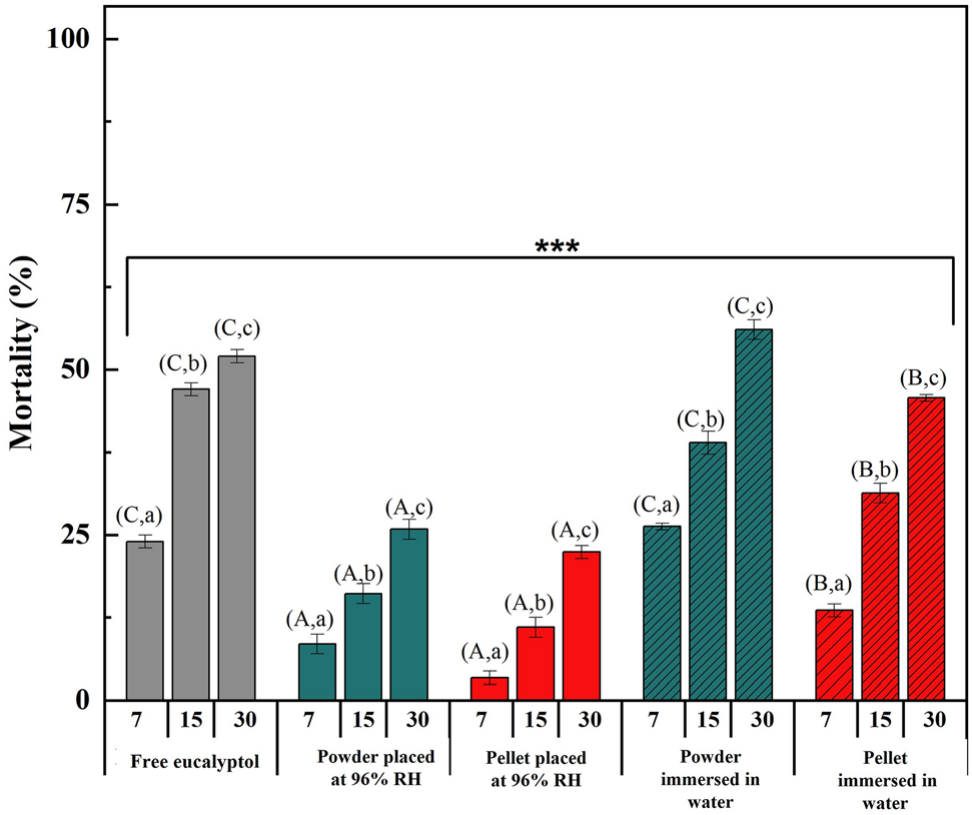
Mortality rates (%) of fifth instar larvae (L_5_) of *Ectomyelois ceratoniae* exposed to free and encapsulated eucalyptol (different letters indicate significant difference in percent mortality for each treatment (uppercase letters) and the exposure period (lowercase letters) Duncan's test at *P* < 0.05).

Fumigation experiments were performed at the LC_50_ for free or encapsulated eucalyptol. The inclusion complex prepared at 1:1 molar ratio was selected for the test due to its better LC value and EE percentage. The insecticidal activity of solid powder or pellets was tested either in a controlled RH environment or immersed in water. The mortality rates of *Ectomyelois ceratoniae* L_5_ were determined at different time intervals (7, 15 and 30 days) in comparison to free eucalyptol (Fig. 5).

The results showed that for all the treatments, toxicity increased with increasing exposure period. For both powder and pellets of inclusion complex placed in a controlled humidity environment (96% RH), a lower toxicity was detected in comparison to free eucalyptol. After 30 days of exposure, free eucalyptol resulted in 53% mortality while the larval mortality rates observed for the solid β‐CD/eucalyptol inclusion complex in the powder and pellet forms were 26% and 22%, respectively. This could be attributed to the fact that encapsulation in solid state, both in the form of powder or pellet, controls and delays the release of eucalyptol, thereby reducing its fumigant toxicity.

When considering the fumigant toxicity of the inclusion complex in both forms immersed in water, the results showed higher mortality rates than the corresponding form exposed to 96% RH. After a 30‐day exposure period, β‐CD/eucalyptol inclusion complex powder immersed in water showed a mortality rate of 56%. A lower percentage of larval mortality (46%) was observed for the fumigation treatment using pellets of β‐CD/eucalyptol inclusion complex immersed in water. These findings are consistent with those obtained in the release studies and demonstrate that the insecticidal toxicity is due to the fumigant activity of eucalyptol. Also, for both treatment condition (96% RH or immersed in water), the higher activity observed for powder compared to pellets could also be attributed to the higher release rate of eucalyptol from powder (Fig. 3(B),(D)).

Also, even though free eucalyptol has shown appropriate high activity it is not used as the preferred application form because the fact that the handling and storage of this oily product is a challenge. Inclusion complexes, mainly pellets, can provide more suitable dosage forms for easier application. Also, compared to free eucalyptol, the volatility of eucalyptol is suppressed in solid inclusion complexes. The latter will be easier to handle, easier to dose (counting or weighing tablets) and less risky for the farmers as they are not in contact with the volatile irritant oil. From a practical perspective, the farmers can place water tanks, in certain corners of the silo, where they can place the number of CD/eucalyptol pellets needed to ensure effectiveness. The eucalyptol gradually dissolves from the tank and comes into contact with the pest. The farmers can then make repeated applications as the process is very simple, as the process requires the action of just adding tablets to the water tank at regular intervals.

Thus, these results proved that the molecular encapsulation of eucalyptol in β‐CD, in different solid forms, at 1:1 molar ratio can effectively serve as a potential solution for the control of the L_5_ of *Ectomyelois ceratoniae*. Importantly, the results indicated that for an optimal fumigant toxicity, solid inclusion complexes of volatile active agents, such as eucalyptol, should be immersed in water to volatilize.

#### 3.3.3 Median lethal time (LT_50_)

The LT_50_ values of various treatments, including free eucalyptol and its solid β‐CD encapsulated forms, powder and pellets, both placed at 96% RH and immersed in water were determined. Free eucalyptol showed a LT_50_ of 27 days while encapsulated forms in solid powder and pellets placed at 96% RH exhibited LT_50_ values of 51 and 48 days, respectively. When these encapsulated forms were immersed in water, the LT_50_ significantly decreased to 25 days for the powder and 32 days for the pellets. These results showed once again that for an optimal and fast fumigant toxicity solid inclusion complexes should be immersed in water in accordance with fumigation studies.

#### 3.3.4 Adult emergence

**Figure 6:**
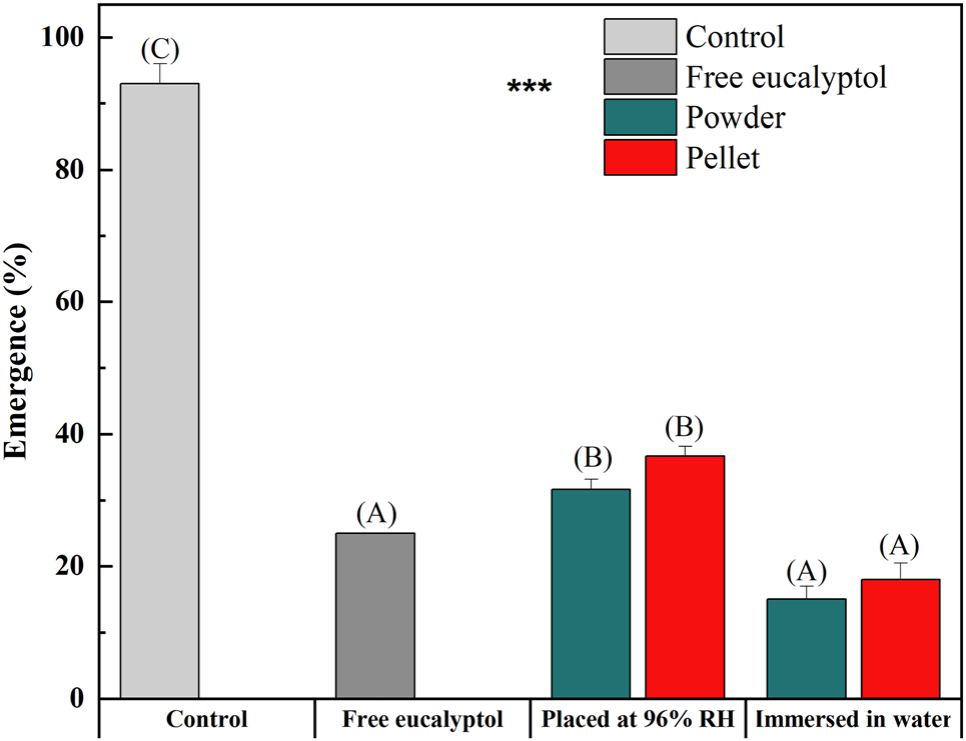
Emergence rate (%) of *Ectomyelois ceratoniae* adults subjected to different treatments (different letters indicate significant differences at *P* < 0.05 in percent emergence for each treatment, Duncan test).

The degree of inhibition of adult emergence of the different treatments was also evaluated after 30 days of storage compared to the control (Fig. 6).

Untreated larvae showed an emergence rate of 93%. Free eucalyptol showed a significant decrease in the percentage of adult's emergence (25%). Treatment with solid powder and pellets of β‐CD/eucalyptol placed at 96% RH reduced the emergence of *Ectomyelois ceratoniae* adults to 32% and 37%, respectively. In addition, immersion of solid inclusion complex in water significantly reduced the emergence of *Ectomyelois ceratoniae* larvae to 15% and 18% for the powder and pellets, respectively. These results showed that eucalyptol itself can affect larval development and prevent adult emergence. The use of β‐CD/eucalyptol inclusion complexes immersed in water resulted in higher emergence inhibition rates. This proved that the new proposed application form could be considered as an effective treatment to control larvae and prevent adult emergence.

#### 3.3.5 Persistence

**Figure 7:**
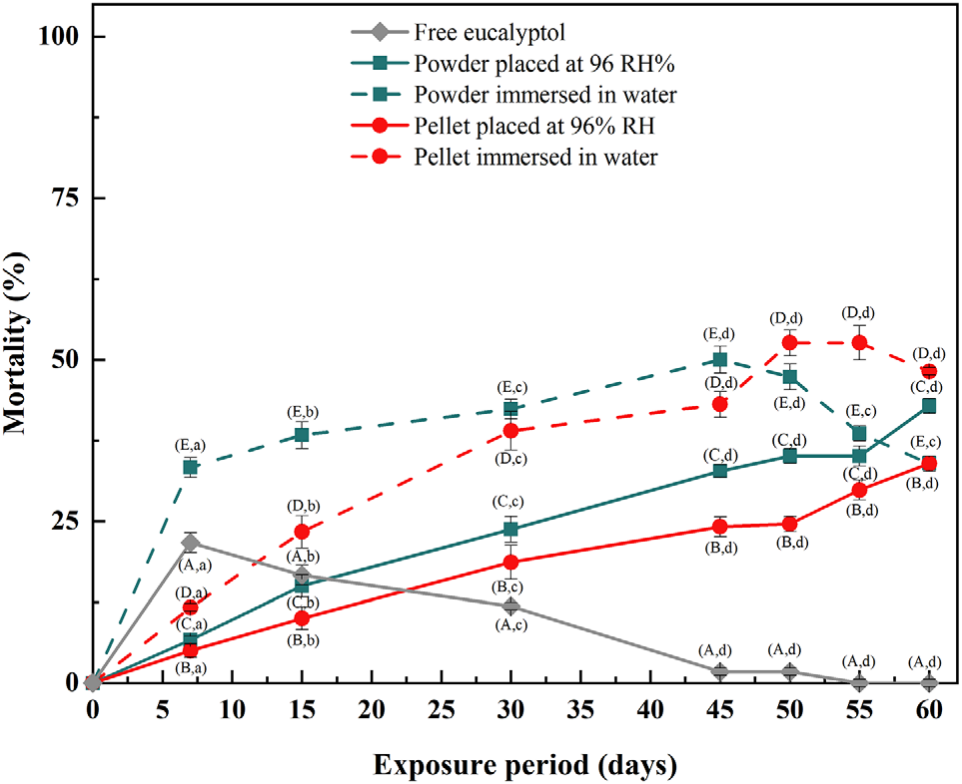
Persistence of the different treatments: free eucalyptol, powder and pellets of solid β‐CD/eucalyptol inclusion complex placed at 96% relative humidity (RH) or immersed in water (different letters indicate significant difference in percent mortality for each treatment (uppercase letters) and the exposure period (lowercase letters) Duncan's test at *P* < 0.05).

The persistence of free and encapsulated eucalyptol was investigated up to 60 days (Fig. 7).

The results showed that the efficacy of the treatment with free eucalyptol, as measured by the larval mortality rates, decreased to almost zero after 55 days. On the contrary, the treatment with all other forms of encapsulated eucalyptol resulted in sustained larval mortality with up to 55 days persistence. Both powder and pellet solid inclusion complexes immersed in water showed the highest mortality rates throughout the study period and thus longer persistence. These observations are once again consistent with the release experiments where inclusion complexes showed delayed release of eucalyptol and thus prolonged larvicidal efficacy.

## 4 CONCLUSION

This study confirmed our hypothesis that encapsulation would result in a more potent and sustained insecticidal activity of eucalyptol. Sustained release of eucalyptol was achieved by formulating β‐CD/eucalyptol inclusion complex in powder or pellet form. Immersion of both powder and pellets in water was necessary to release the full amount of encapsulated guest. Furthermore, the results showed that both powder and pellets immersed in water induced the highest larval mortality and adult emergence inhibition against L_5_ stage, highlighting the effectiveness of this treatment method for immediate larval control. Persistence tests showed that the same application procedure allowed sustained larvicidal activity. Taken together, the results of this study suggest that pelletizing β‐CD/eucalyptol solid inclusion complex could be an efficient formulation technique to improve powder handling, provide single unit dosage forms and offer delayed release of active volatiles. Importantly, for an optimal insecticidal activity, the pellets should be immersed in water to volatilize. This could be considered a promising approach for long‐term management of *Ectomyelois ceratoniae*.

## CONFLICT OF INTEREST

The authors have no conflicts of interest to disclose.

## AUTHOR CONTRIBUTIONS

Sophie Fourmentin: conceptualization, funding acquisition, methodology, project administration, supervision, visualization, writing – review and editing. Jouda Mediouni Ben Jemâa: conceptualization, funding acquisition, methodology, project administration, supervision, visualization. Miriana Kfoury: conceptualization, methodology, supervision, visualization, formal analysis, writing – original draft, writing – review and editing. Hadhami Chargui: investigation, data curation, writing – original draft. Abir Soltany: writing – original draft. Tasnim Djebbi: methodology. Soumaya Haouel Hamdi: formal analysis.

## Data Availability

The data that support the findings of this study are available from the corresponding author upon reasonable request.
